# Defined Extracellular Matrix Contexts Rewire 3D Breast Cancer Cell Models: Effects of Sulfated Hyaluronan in the Expression of Matrix Effector and Matrix-Based Cell Functional Properties

**DOI:** 10.3390/cancers18203288

**Published:** 2026-10-12

**Authors:** Marios Vetoulas, Maria Drosinou, Nikoleta Alexiou, Angeliki Farmaki, Zoi Piperigkou, Nikos K. Karamanos

**Affiliations:** Biochemistry, Biochemical Analysis & Matrix Pathobiology Research Group, Laboratory of Biochemistry, Department of Chemistry, University of Patras, 26504 Patras, Greecezoipip@upatras.gr (Z.P.)

**Keywords:** breast cancer, triple-negative breast cancer, extracellular matrix, tumor microenvironment, 3D spheroids, collagen type I, fibronectin, hyaluronan, sulfated hyaluronan, CD44, RHAMM, HAS2, integrins

## Abstract

**Background:** Breast cancer progression is strongly influenced by tumor cell interactions with the extracellular matrix (ECM). Three-dimensional (3D) cell culture models provide more relevant systems for studying aspects of the tumor microenvironment (TME), including tumor architecture, matrix remodeling and cell-ECM communication. Collagen type I (Col I), fibronectin (FN) and hyaluronan (HA) are key ECM components involved in breast cancer progression. At the same time, integrins act as major matrix-sensing receptors that translate ECM cues into cellular responses. In this study, we investigated how defined ECM contexts regulate functional cell properties in shERβ MDA-MB-231 and Hs578T advanced 3D breast cancer cell models. **Methods:** Cells were cultured in 2D and 3D systems under matrix-free conditions or in Col I, FN, and mixed Col I/FN environments, in the presence or absence of HA or sulfated HA (sHA). Spheroid growth, matrix-dependent spheroid organization and dissemination were evaluated. In parallel, selected collagen- and fibronectin-associated integrin subunits, together with HA-related molecules including HAS2, CD44, and RHAMM, were analyzed to assess receptor-mediated matrix- and HA-associated responses. **Results:** Matrix substrates strongly influenced breast cancer spheroid behavior. Col I promoted outward dissemination, whereas FN-containing environments favored compact organization and restricted spreading. HA supported spheroid-like organization in a matrix-dependent manner, whereas sHA disrupted organized spheroid assembly in ECM-rich conditions, resulting in looser structures or smaller cell colonies, depending on the matrix context. Gene expression analysis revealed model-dependent remodeling of matrix- and HA-associated programs, with broader adhesion-related responses in shERβ MDA-MB-231 cells and stronger dimensionality-driven regulation in Hs578T cells. **Conclusions:** Overall, these findings demonstrate that defined ECM contexts actively regulate breast cancer spheroid behavior and matrix-associated receptor programs. Col I, FN and mixed Col I/FN environments generated distinct 3D phenotypes, affecting spheroid growth, organization and dissemination, while HA/sHA further modified these matrix-dependent responses. This study supports sHA as a functional matrix molecule with anti-cancer relevance and highlights the value of 3D matrix-based models for investigating tumor–matrix communication in breast cancer.

## Simple Summary

Breast cancer cells growth and spreading are strongly influenced by the surrounding extracellular matrix, which provides structural and biochemical signals. In this study, we used three-dimensional breast cancer cell models to better mimic features of the tumor microenvironment and examine how specific major matrix components such as collagen type I, fibronectin and hyaluronan affect cancer cell behavior. The effects of the chemically modified form of hyaluronan, the sulfated hyaluronan, were evaluated in matrix-free and matrix-based niches. Our findings show that defined matrix environments generated distinct three-dimensional spheroid phenotypes, affecting growth, organization and dissemination. Collagen type I promoted outward spreading of spheroid-derived cells, fibronectin environments favored compact organization and restricted dissemination, whereas sulfated hyaluronan further disrupted spheroid organization under specific matrix conditions. These results support the use of three-dimensional matrix-based models to study breast cancer behavior and suggest that sulfated hyaluronan may be further explored as a bioactive molecule with potential anti-cancer relevance.

## 1. Introduction

Breast cancer remains one of the most common malignancies worldwide and represents a major cause of cancer-related morbidity and mortality among women [1]. Despite advances in early diagnosis, molecular classification and targeted therapeutic strategies, disease recurrence, therapy resistance and metastatic dissemination continue to limit clinical outcome [2]. Breast cancer progression is not determined only by cancer-cell intrinsic genetic and epigenetic alterations but is strongly shaped by dynamic interactions with the surrounding TME [3].

The TME is a complex and continuously remodeled network composed of stromal cells, immune cells, soluble mediators and ECM components [4]. Within this network, the ECM has emerged as a central regulator of tumor behavior, rather than a passive structural scaffold [5,6]. Through biochemical, mechanical and spatial cues, the ECM regulates cancer cell survival, proliferation, adhesion, migration, invasion and response to therapy [7]. During breast cancer progression, ECM homeostasis becomes progressively disrupted, resulting in altered matrix deposition, fiber organization and tissue stiffness [8]. These remodeled matrix properties modify cancer cell mechanosensing and downstream signaling, thereby supporting more aggressive phenotypes, including enhanced survival, motility and metastatic potential [9,10].

3D culture models have become valuable tools for studying TME interactions. In contrast to conventional two-dimensional (2D) monolayers, 3D spheroids better reproduce aspects of tumor architecture, including cell–matrix interactions, spatial organization and matrix-associated signaling [11,12,13]. These models are particularly useful for investigating ECM remodeling, receptor-mediated matrix sensing and functional cancer properties such as spheroid growth, compaction, dissemination and treatment response [14]. Therefore, 3D systems provide a more relevant experimental framework for examining how breast cancer cells behave within matrix-associated contexts [15].

Triple-negative breast cancer (TNBC) is an aggressive breast cancer subtype characterized by the absence of estrogen receptor alpha (ERα), progesterone receptor (PR) and HER2 expression. TNBC is commonly associated with increased recurrence risk, metastatic potential and limited targeted treatment options [16]. Importantly, TNBC progression is strongly linked to ECM remodeling, epithelial-to-mesenchymal plasticity, altered adhesion programs and invasive behavior. Previous characterization of shERβ MDA-MB-231 and Hs578T spheroids supported the relevance of these models for studying ECM-associated dynamics and functional cancer properties under 3D conditions [14,17]. The shERβ MDA-MB-231 cell model is a stably transfected, ERβ-suppressed clone of the parental MDA-MB-231 cell line that has been previously characterized by our group and used in this study as a defined model to investigate ECM-dependent responses under conditions of reduced ERβ expression [18].

Among ECM components, Col I and FN are major regulators of breast cancer cell behavior [19]. Col I is one of the most abundant fibrillar ECM proteins and is frequently increased during tumor-associated matrix remodeling. Enhanced collagen deposition, alignment and cross-linking contribute to matrix stiffening and altered tissue mechanics, promoting mechanotransduction and invasive behavior [20]. On the other hand, FN is an adhesive ECM glycoprotein involved in matrix organization, cell attachment and migration [21]. Increased FN deposition in tumors is associated with altered cell–matrix communication supporting tumor progression and therapy resistance [5]. Together, Col I and FN actively shape the physical and biochemical properties of the tumor matrix and influence how cancer cells organize, spread and disseminate.

In addition to fibrillar and adhesive ECM proteins, HA is a native non-sulfated glycosaminoglycan involved in tumor-matrix communication. HA contributes to tissue hydration, matrix organization, inflammation and cell motility [21]. Its biological activity depends on molecular size, synthesis, degradation and receptor-mediated signaling. HA synthase 2 (HAS2) is a major enzyme involved in HA production, while CD44 and RHAMM are key HA-associated receptors that regulate cell function and tumor progression [22,23]. Dysregulation of the HA-HAS2-CD44/RHAMM axis has been linked to breast cancer aggressiveness and altered communication between cancer cells and the ECM [24,25].

Sulfated hyaluronan (sHA) represents a chemically modified HA derivative with distinct biological properties compared with the non-sulfated HA. Although native HA is frequently associated with tumor-promoting processes, sulfation alters its physicochemical properties and may modify its interactions with HA-binding proteins, receptors and matrix components [26,27]. Previous studies have suggested that sHA may exert anti-cancer-related effects, including modulation of tumor cell behavior and interference with pro-tumorigenic signaling [27]. Such effects have been reported not only in breast cancer but also in other tumor models, including prostate and lung cancer, although responses appear to depend on tumor type and cellular context [27,28]. However, its impact on breast cancer spheroid organization within defined ECM environments remains insufficiently explored.

Integrins are central mediators of cell-ECM communication and provide a direct mechanistic link between ECM composition and cancer cell behavior [29]. These transmembrane receptors recognize specific matrix ligands, including Col I and FN and transmit extracellular cues into intracellular adhesion and signaling programs [30,31]. Through focal adhesion formation and downstream pathways involving cytoskeletal remodeling and mechanotransduction, integrins regulate cell attachment, spreading, migration and survival [32,33]. Therefore, integrin-associated matrix sensing represents a key process through which breast cancer cells adapt to distinct ECM environments [30,31].

Considering these data, we investigated how defined ECM substrates regulate breast cancer cell behavior in 2D and 3D culture systems using shERβ MDA-MB-231 and Hs578T breast cancer models. Our central hypothesis was that breast cancer spheroid phenotype is jointly determined by ECM composition and HA sulfation, through context-dependent modulation of matrix-sensing programs. We therefore examined the effects of Col I, FN and mixed Col I/FN environments, with or without HA and sHA, on spheroid growth, matrix-dependent organization and dissemination. In parallel, selected integrin subunits and HA-related mediators, including *HAS2*, *CD44* and *RHAMM*, were analyzed to determine whether the observed functional phenotypes were accompanied by changes in matrix- and HA-associated transcriptional programs. Bioinformatic analyses were used as complementary support for the biological relevance of the selected matrix-associated genes. This work aims to clarify how major matrix substrates, HA sulfation and receptor-mediated sensing collectively shape breast cancer spheroid architecture and matrix-dependent behavior.

## 2. Materials and Methods

### 2.1. HA and sHA

The HA fragments of ~50 kDa were isolated at Fidia Farmaceutici S.p. As described in a previous study of our research group [30]. The sulfated HA (MW~50 kDa, degree of sulfation: 2.8) was kindly provided by Fidia Farmaceutici S.p.A. (Abano Terme, Italy). The selection of approximately 50 kDa HA and sHA and the treatment concentration of 200 µg/mL was based on previous experimental studies from our research group using these conditions in breast cancer cells [30]. The use of non-sulfated HA and sHA of the same molecular weight allowed the effects associated with sulfation to be examined under matched molecular-size conditions.

### 2.2. Cell Cultures and Reagents

The shERβ MDA-MB-231 cell line resulted from stable transfections of parental MDA-MB-231 breast cancer cells (HTB-26, ATCC, Manassas, VA, USA) with shRNA lentiviral particles targeting *ESR2*, and has been previously characterized by our research group [18]. Hs578T cells (HTB-126, ATCC, Manassas, VA, USA) were used as an additional TNBC model. Both cell lines were incubated in monolayer cultures at 37 °C in an atmosphere of 5% CO_2_ and 95% humidified atmospheric air. The cell culture medium utilized was Dulbecco’s modified Eagle’s medium (DMEM; LM-D1110/500, Biosera, Cholet, France), enriched with 10% fetal bovine serum (FBS; Capricorn Scientific, Ebsdorfergrund, Germany), as well as with antimicrobial agents (100 IU/mL penicillin, 100 μg/mL streptomycin, 10 μg/mL gentamycin sulfate, and 2.5 μg/mL amphotericin B, Biosera, Cholet, France), and 2 mM L-glutamine (Biosera, Cholet, France). The cells were recultured after trypsin-EDTA 1X was diluted in PBS (LM-T1706; Biosera, Cholet, France) to 80–85% confluency.

### 2.3. 3D Cell Cultures and Spheroid Development

BC cell-derived 3D spheroids were formed by incubating cancer cells, at 37 °C in an atmosphere of 5% CO_2_ and 95% humidified atmospheric air in 96-well round-bottom plates with ultra-low adhesive properties (SPL Life Sciences, Pocheon-si, Republic of Korea). Specifically, shERβ MDA-MB-231 and Hs578T cells were seeded at a density of 15,000 cells per well and incubated for 72 h in complete medium (DMEM 10% FBS) without any medium changes, allowing spheroid development and formation. Spheroid development was monitored through phase-contrast microscopy.

### 2.4. RNA Extraction, cDNA Synthesis and Real-Time qPCR

For total RNA isolation from 2D cell cultures incubated at 37 °C in an atmosphere of 5% CO_2_ and 95% humidified atmospheric air, shERβ MDA-MB-231 and Hs578T cells were seeded at a density of 600,000 cells per 60 mm cell culture dish in 10% FBS-enriched DMEM medium for 48 h until reaching 70–75% confluence. Subsequently, the medium was replaced with serum-free medium, and cells were serum-starved for 16–20 h. Then the cells were treated with ~50 kDa HA and sHA, respectively, for 24 h at a final concentration of 200 μg/mL. To harvest the cells, 1X trypsin-EDTA in PBS was used, followed by two washes with cold PBS 1X, each performed by centrifugation at 3000 rpm for 3 min. Total RNA isolation was performed using NucleoSpin^®^RNA Plus Kit (Macherey-Nagel, Dueren, Germany), following the manufacturer’s instructions. For total RNA isolation from 3D cell cultures, cell-derived spheroids were cultured for 72 h, starved for 16–20 h, and then treated as in 2D cultures. The duration of starvation was selected based on prior literature and preliminary optimization experiments to ensure sufficient pathway quiescence without compromising cell viability or spheroid integrity. A total of 48 spheroids per condition (control, ~50 kDa, and sHA), each seeded with 15,000 cells, were collected for RNA extraction. Afterward, spheroids were collected and their total RNA was isolated as described above, except that Trypsin EDTA solution was not used, because spheroids are not attached to any surface; instead, spheroids were harvested using a 1000 μL pipette tip. Quantification of total isolated RNA was performed by measuring absorbance at 260 nm with a photometer, and purity was assessed by evaluating the 260/280 and 260/230 ratios for all RNA extracts. Total RNA from both 2D and 3D cultures was reverse-transcribed, using the PrimeScript 1st strand cDNA synthesis kit (RR037A, Takara Bio Inc., Goteborg, Sweden), perfect real-time. Real-time qPCR analysis was conducted according to the manufacturer’s guidelines, and the Rotor Gene Q (Qiagen, Germantown, MD, USA) was used for amplification. To further ensure the absence of DNA contamination, non-template controls (NTCs) were included in all qPCR reactions and melt curve analyses were performed to confirm the specificity of the amplified products. An NTC sample, containing master mix reagents and primers, but replacing cDNA with PCR-grade water, was included as a negative control. The reactions were performed in duplicate, with a standard curve for each primer pair to validate the assay. Additionally, we performed a melting curve analysis to detect the Maxima SYBR Green/ROX qPCR Master Mix (2X) enzyme (Thermo Fisher Scientific, Waltham, MA, USA). The fluorescence threshold, set above the background, was used to determine the threshold cycle (Ct) number, representing the point of product accumulation during the early logarithmic phase of amplification. Subsequently, the relative expression of gene transcripts was calculated using the ∆∆Ct method, in which the Ct of each gene of interest was normalized to the Ct of the housekeeping gene 18S rRNA. Among other normalizers (e.g., GAPDH), 18S rRNA was selected as the reference gene for normalization due to its stable expression across treatment conditions in our experimental system. Fold changes (arbitrary units) were determined as 2^−∆∆Ct^. Detailed information about the target genes and the primers used is provided in Table 1.

### 2.5. Spheroid Growth Assay in HA/sHA-Treated Spheroids

10,000 cells per well of shERβ MDA-MB-231 and Hs578T cells were seeded in 96-well round-bottom ultra-low adhesion plates (SPL Life Sciences, Pocheon-si, Republic of Korea). Until spheroids formed, cells were cultivated at 37 °C in an atmosphere of 5% CO_2_ and 95% humidified atmospheric air for three days in an enriched medium without any media changes. The cells were then starved for 16–20 h in a serum-free medium and then treated with HA (~50 kDa) and sHA at a final concentration of 200 μg/mL. Photos were taken at every step using a phase-contrast microscope to monitor spheroid development and growth in the presence of HA. Finally, retreatments were performed every 48 h to mimic the in vivo experiments previously conducted by our lab, at the same final concentration.

### 2.6. Spheroid Dissemination Assay in HA/sHA-Treated Spheroids

10,000 cells per well of shERβ MDA-MB-231 and Hs578T cells were seeded in 96-well round-bottom ultra-low adhesion plates (SPL Life Sciences, Pocheon-si, Republic of Korea). Until spheroids formed, cells were cultivated at 37 °C in an atmosphere of 5% CO_2_ and 95% humidified atmospheric air for three days in an enriched medium without any media changes. The cells were then starved for 16–20 h in a serum-free medium and then treated with HA (~50 kDa) and sHA at a final concentration of 200 μg/mL for 24 h. Spheroids were then transferred in 96-well flat bottom plate. Photos were taken at every step using a phase-contrast microscope to monitor spheroid dissemination capacity in the presence of HA.

### 2.7. Matrix-Based Spheroid Growth Assay in HA/sHA-Treated Spheroids

Matrix-based spheroid growth assays were performed using Col I, FN and HA- or sHA-containing matrix combinations. FN was diluted in sterile PBS 1X to prepare aliquots at an intermediate concentration of 100 μg/mL and stored at −20 °C until use. Sterile Col I was stored at −20 °C and diluted in sterile 0.01 N HCl to prepare an intermediate stock concentration of 10 mg/mL. Collagen solutions were handled gently at 4 °C to avoid premature gelation and mild vortexing was applied only when necessary to ensure complete dissolution.

For matrix preparation, Col I and FN were used at final concentrations of 2 mg/mL and 5 μg/mL, respectively. Cells were harvested using trypsin-EDTA 1X in PBS and resuspended in a complete culture medium containing 10% FBS. A cell suspension master mix was prepared at a concentration of 200,000 cells/mL and kept on ice throughout the procedure.

To prepare collagen-containing matrix solutions, Col I was mixed with sterile NaOH 1 N for neutralization and HEPES 1 M, pH 7.4, to obtain an intermediate collagen concentration of 4 mg/mL. For FN matrices, FN was diluted in HEPES-containing buffer to an intermediate concentration of 10 μg/mL. For mixed Col I/FN matrices, Col I and FN were combined to obtain intermediate concentrations of 4 mg/mL and 10 μg/mL, respectively. All matrix solutions were prepared on ice.

For HA- or sHA-containing matrices, HA or sHA fragments of approximately 50 kDa were used. HA and sHA were resuspended in sterile PBS to prepare stock solutions of 2 mg/mL. For matrix incorporation, HA or sHA was added to the Col I/FN mixture to obtain an intermediate concentration of 0.4 mg/mL, corresponding to a final concentration of 0.2 mg/mL after dilution with the cell suspension. The final matrix combinations used for spheroid formation were Col I, FN, Col I/FN, and Col I/FN supplemented with HA or sHA.

After preparation, each matrix solution was mixed gently at a 1:1 ratio with the cell suspension, avoiding bubble formation. The final concentrations in the cell–matrix mixtures were 2 mg/mL Col I, 5 μg/mL FN and, where applicable, 0.2 mg/mL HA or sHA. These concentrations were used to establish reproducible, experimentally defined ECM conditions for comparative analysis and were not intended to quantitatively reproduce the absolute concentrations of these components within the breast tumor microenvironment *in vivo*.

The cell–matrix mixtures were then transferred into ultra-low adhesion 96-well plates at 50 μL per well.

The plates were incubated for 2 h at 37 °C and 5% CO_2_ to allow gel formation. After gelation, 200 μL of complete culture medium containing 10% FBS was carefully added to each well. Spheroid formation and growth were monitored over time using phase-contrast microscopy.

For matrix preparations that did not contain Col I, NaOH was omitted. When HA or sHA was used in different matrix combinations, HEPES volumes were adjusted accordingly to maintain the final volume and concentration of the scaffold mixture.

### 2.8. Matrix-Based Dissemination Assay

Cells were seeded at a density of 10.000/well and spheroids were formed as described in Section 2.3.

A flat-bottom 96-well plate was coated with Col I and/or FN at the concentrations described in Section 2.7. The gels were formed as described above, with the only difference being in the final mixing of substrate solution. In this case, substrate solutions were mixed with equal volumes of serum-plus medium instead of the cell’s master-mix. The flat bottom plate was incubated for 2 h at 37 °C and 5% CO_2_ for gel formation.

The spheroids were transferred from the ultra-low-adhesion plate to the plate with the coatings using 1000 μL tips at a density of 1 spheroid/well, and photos were taken at 24, 48, and 72 h.

### 2.9. Matrix-Based Dissemination Assay with Incorporation of HA/sHA

Hs578T cells were seeded in an ultra-low adhesion 96-well plate at a density of 10,000 cells/well as described in Section 2.3. The treatment with HA and sHA was performed in two different ways. The one way was with the treatment incorporated into the gel with Col I or/and FN and the other way was that the cells were treated with HA or sHA at first and the next day they were transferred into the gel. For the cells that the treatment of HA and sHA was incorporated into the gel, the concentrations and the methodology were performed as described in Section 2.7 and Section 2.8. For the cells that were pre-treated, a flat-bottom 96-well plate was coated with Col I and/or FN at final concentrations as described in Section 2.7 and the treatment of HA and sHA was performed as described in Section 2.6. For all experiments, spheroid development was monitored through phase contrast microscopy for 0 h, 24 h, 48 h, and 72 h.

### 2.10. Kaplan–Meier Survival Analysis Bioinformatics Tool

Kaplan–Meier Plotter (https://kmplot.com/analysis, assessed on 15 June 2025) is an online survival analysis tool that assesses the prognostic significance of gene expression across various cancers, including BC. By integrating transcriptomic data from public sources such as TCGA, GEO, and EGA, researchers can generate survival plots based on the expression levels of individual genes or gene combinations. Users can stratify patients by median, tertiles, or quartiles of expression and assess outcomes such as overall survival, relapse-free survival (RFS), or distant metastasis-free survival (DMFS). This facilitates the identification of candidate biomarkers associated with patient prognosis and supports hypothesis-driven exploration of gene function in clinical contexts. In this study, several genes of interest (e.g., *ITGA1*, *ITGA2*, *ITGB1*, etc.) were queried. The “Breast Cancer” dataset was selected. Patient stratification was based on a median split (high vs. low expression), and the endpoint analyzed was RFS. The resulting plots provided hazard ratios (HRs), confidence intervals (CIs), and log-rank *p*-values to evaluate the prognostic relevance of gene expression levels.

### 2.11. The STRING Database

The STRING database (https://string-db.org, assessed on 15 June 2026) is part of the ELIXIR Core Data Resources and is a well-known resource for predicting protein–protein interactions. These interactions include both direct (physical) and indirect (functional) associations, derived from computational predictions, knowledge transfer between organisms, and interactions compiled from other primary databases. The five main sources of data are (a) Genomic Context Predictions, (b) High-throughput Laboratory Experiments, (c) (Conserved) Co-expression, (d) Automated Text Mining, and (e) Previous Knowledge in Databases. The interactions presented in the STRING network may represent either known associations supported by published experimental evidence or predicted interactions inferred through computational methods, such as text mining, co-expression analysis, or protein homology. The process followed in this study was as follows: selecting the “Multiple proteins” tool, entering the protein names and the studied organism, and then navigating to the Legend section.

### 2.12. Statistical Analysis

Statistical significance was assessed using *t*-tests and multiple *t*-tests to identify differences between the various groups, for both 2D and 3D models (control, HA, and sHA). The experiments were performed in triplicate (*n* = 3). Simple *t*-tests were used for individual pairwise comparisons, whereas multiple *t*-tests were analyzed using the Holm–Šídák method to correct for multiple comparisons. A *p*-value ≤ 0.05 was considered statistically significant, with asterisks (*) indicating significant differences between HA- and sHA-treated cells relative to their untreated counterparts, which served as the control. All statistical analyses and graphs were made using GraphPad Prism version 8.0.1 (GraphPad Software, Boston, MA, USA).

Spheroid area was quantified in mm^2^ from phase-contrast microscopy images using ImageJ software version 1.50b (Launcher Symmetry Software, LOCI, University of Wisconsin, Madison, WI, USA). Image scale was calibrated separately for the 4× and 10× objectives using the known grid distance of a Neubauer hemocytometer. The lower magnification was used when a wider field of view was required to capture the complete spheroid or dissemination area, whereas the higher magnification was used when the entire structure could be adequately visualized within a smaller field. Following scale calibration, spheroid outlines were manually defined, and the corresponding areas were measured in calibrated units (mm^2^).

## 3. Results

### 3.1. HA/sHA-Dependent Remodeling of Integrin and HA-Associated Gene Expression in 2D and 3D Breast Cancer Models

To assess whether HA and sHA modulate matrix- and HA-associated transcriptional programs, real-time qPCR analysis was performed in 2D cultures and 3D spheroids of shERβ MDA-MB-231 and Hs578T cells. The analysis included selected collagen- and fibronectin-associated integrin subunits, namely *ITGA1*, *ITGA2*, *ITGAV*, *ITGB1*, *ITGB3* and *ITGB5*, together with HA-related mediators, including *HAS2*, *CD44* and *RHAMM*. Expression values were normalized to the corresponding untreated 2D control condition, allowing the effects of culture dimensionality and HA/sHA treatment to be evaluated.

**Figure 1:**
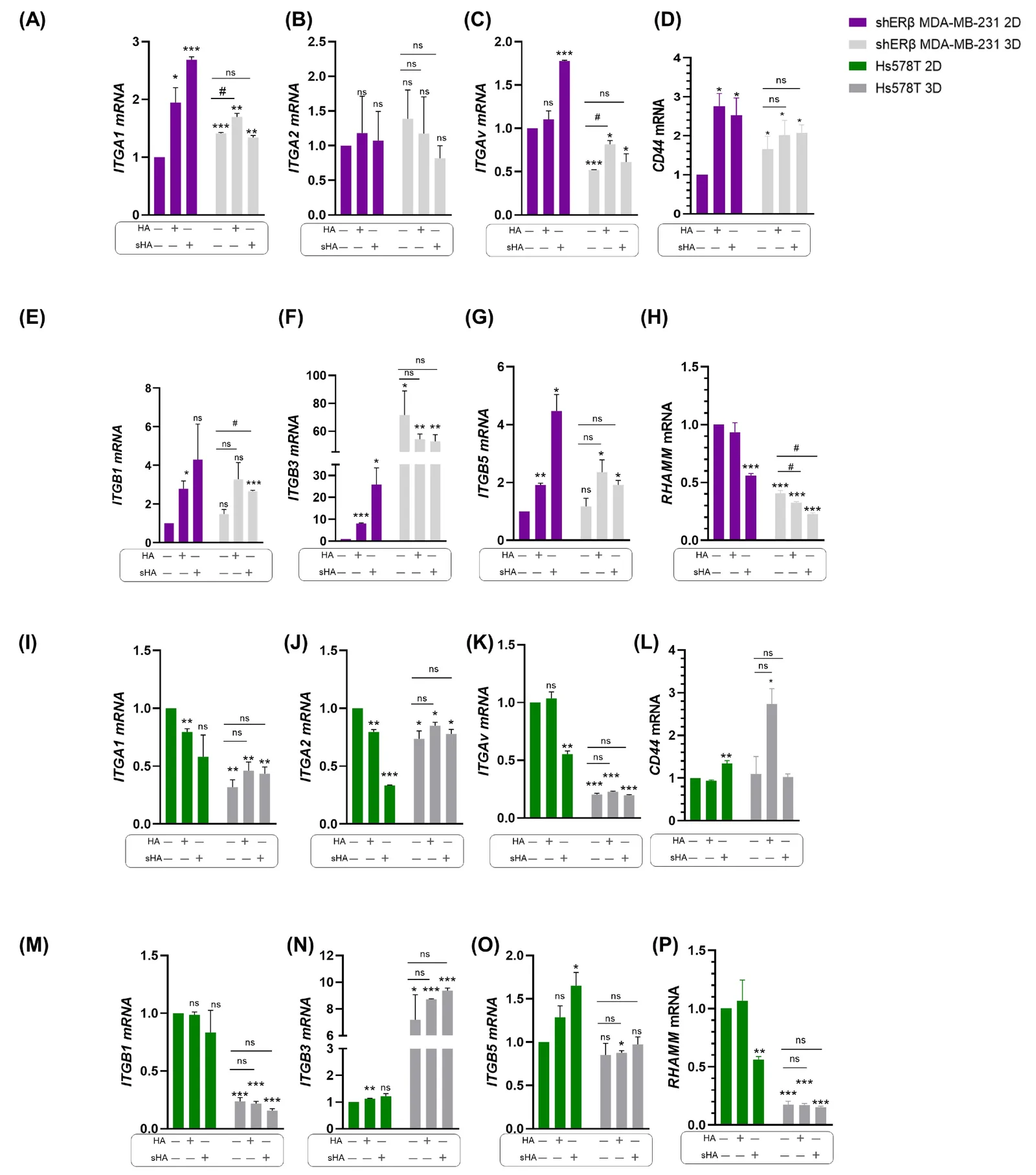
Real-time qPCR analysis of selected integrin subunits and HA-associated genes in 2D monolayers and 3D spheroids following treatment with HA or sHA. (**A**) *ITGA1*, (**B**) *ITGA2*, (**C**) *ITGAV*, (**D**) *CD44*, (**E**) *ITGB1*, (**F**) *ITGB3*, (**G**) *ITGB5*, (**H**) *RHAMM*; graphs (**A**–**H**) refer to the shERβ MDA-MB-231 cell line. (**I**) *ITGA1*, (**J**) *ITGA2*, (**K**) *ITGAV*, (**L**) *CD44*, (**M**) *ITGB1*, (**N**) *ITGB3*, (**O**) *ITGB5*, (**P**) *RHAMM*; graphs (**I**–**P**) refer to the Hs578T cell line. Data are presented as mean ± SD from triplicate experiments (*n* = 3). An asterisk (*) indicates statistically significant differences (*p* < 0.05), two asterisks (**) indicate statistically significant differences (*p* < 0.01), while three asterisks (***) indicates statistically significant differences (*p* < 0.001) compared to 2D control groups. Statistically significant differences (*p* < 0.05) are indicated by a hash (#), while none statistically significant differences are indicated with (ns).

In shERβ MDA-MB-231 cells, HA- and sHA-treated conditions induced significant differences in integrin expression. *ITGA1* was increased across several conditions, with sHA inducing a clear upregulation in 2D cultures (Figure 1A). *ITGA2* expression showed no major consistent alteration (Figure 1B). In contrast, *ITGAV* displayed condition-dependent regulation, being upregulated following sHA treatment in 2D cultures but reduced under 3D conditions compared with the 2D control (Figure 1C). These findings suggest that culture dimensionality modifies the response of α-integrin subunits in response to HA/sHA treatments.

Regarding β-integrin subunits, *ITGB1* was increased after HA treatment in 2D cultures, whereas sHA specifically induced *ITGB1* expression in 3D spheroids compared with both 2D and untreated 3D controls (Figure 1E). *ITGB3* and *ITGB5* showed broader upregulation across several conditions. *ITGB3* was strongly increased in 3D spheroids and responded to sHA in 2D cultures, suggesting activation of β3-associated adhesion programs (Figure 1F). *ITGB5* was also upregulated in response to HA-derived treatments, indicating that shERβ MDA-MB-231 cells maintain an active integrin response across both 2D and 3D culture conditions (Figure 1G).

The HA-associated receptors showed a distinct expression pattern in shERβ MDA-MB-231 cells. *RHAMM* expression was reduced after sHA treatment in 2D cultures and was also reduced in 3D spheroids compared with the control (Figure 1H). Notably, sHA treatment in 3D further decreased *RHAMM* expression, suggesting that both HA sulfation and 3D organization negatively regulate *RHAMM* in this model (Figure 1H). In contrast, *CD44* was upregulated after sHA treatment in 2D cultures and was also increased in 3D spheroids compared with the 2D control (Figure 1D). However, HA or sHA treatment did not induce further major changes among the 3D conditions, suggesting that *CD44* expression is mainly influenced by sHA in 2D and by the transition to 3D culture (Figure 1D).

In Hs578T cells, the integrin response was mainly driven by culture dimensionality. *ITGA1*, *ITGA2*, *ITGAV* and *ITGB1* were downregulated in 3D spheroids compared with the 2D control, and this pattern was maintained after HA and sHA treatment without further major changes among the 3D conditions (Figure 1I–K,M). *ITGA2* was also reduced after sHA treatment in 2D cultures (Figure 1J). In contrast, *ITGB3* showed the opposite pattern, being upregulated in 3D spheroids and remaining elevated after HA or sHA treatment (Figure 1N). *ITGB5* showed more limited condition-dependent changes, with sHA inducing a moderate increase in 2D cultures (Figure 1O).

Regarding HA-associated receptors in Hs578T cells, *CD44* showed only a slight increase after sHA treatment in 2D cultures (Figure 1L). The transition to 3D spheroids did not markedly alter *CD44* expression, and HA or sHA treatment did not produce further changes under 3D conditions (Figure 1L). *RHAMM* was downregulated after sHA treatment in 2D cultures and showed further reduction in 3D spheroids. However, HA or sHA treatment did not cause additional *RHAMM* modulation among the 3D conditions (Figure 1P).

Overall, real-time qPCR analysis revealed distinct model-dependent responses. shERβ MDA-MB-231 cells displayed broader HA/sHA-responsive remodeling of adhesion-related integrins, together with opposite regulation of *CD44* and *RHAMM*. In contrast, Hs578T cells showed a stronger dimensionality-driven transcriptional response, characterized by downregulation of several collagen- and fibronectin-associated integrins and persistent induction of *ITGB3* in 3D spheroids. These results indicate that HA sulfation and 3D culture dimensionality differentially regulate integrin-associated matrix sensing and HA-related receptor programs in breast cancer models.

### 3.2. Bioinformatics Analysis of HA-Associated and Integrin-Related Genes in Breast Cancer

Bioinformatics analysis was performed to evaluate the prognostic relevance and interaction profile of selected HA-associated and integrin-related genes in breast cancer. Kaplan–Meier survival analysis was used to assess the association of *ITGB1*, *ITGAV*, *ITGB3*, *CD44,* and *RHAMM* expression with patient outcome in Luminal A and TNBC cohorts. In parallel, STRING analysis was used to examine the functional interaction network among HA-associated receptors, integrin subunits, and ECM-related binding partners.

**Figure 2:**
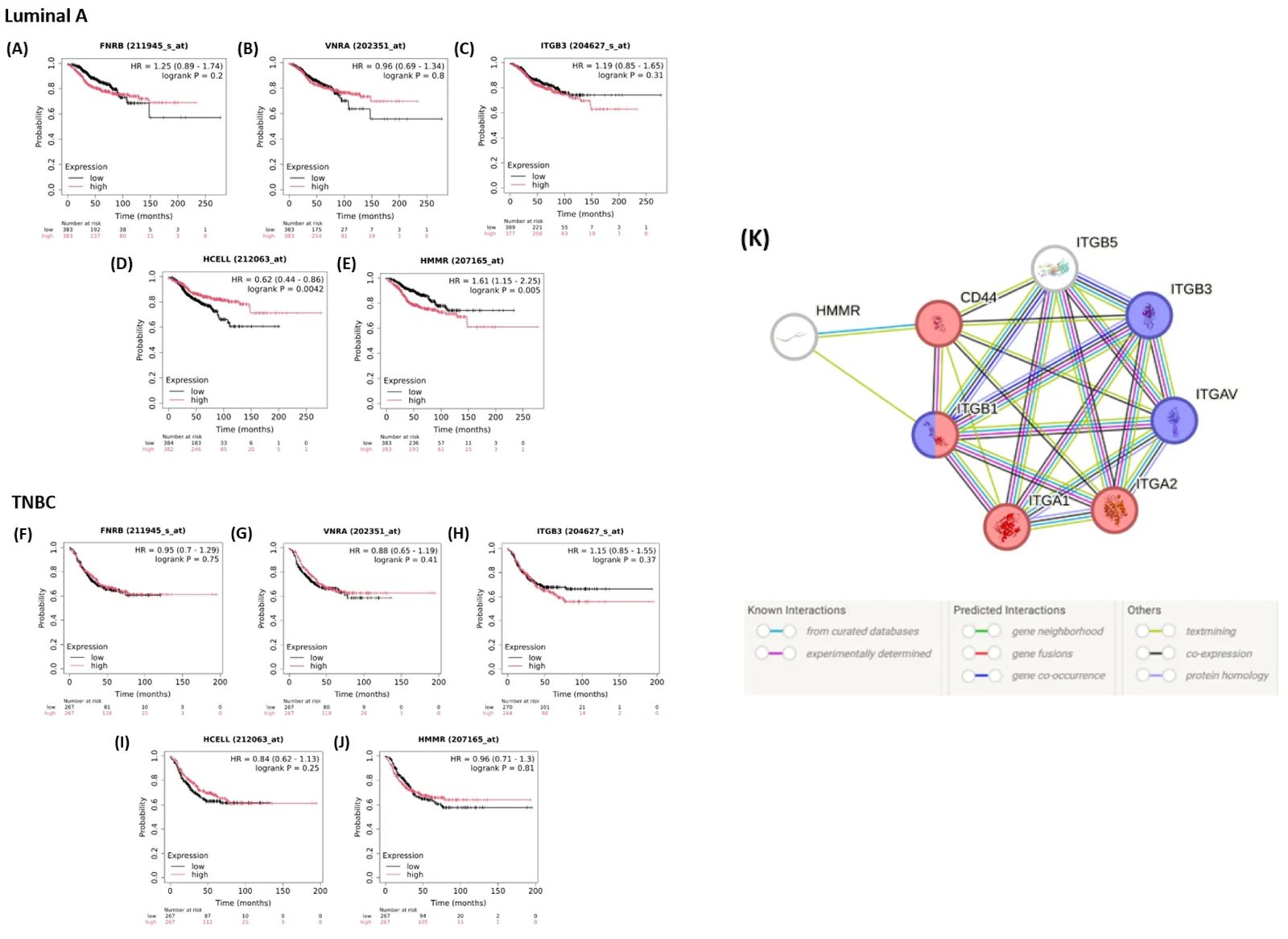
Prognostic (survival) and functional network analysis of HA-associated and integrin-related genes. Luminal A plots include (**A**–**E**), whereas TNBC plots include (**F**–**J**). *p*-value and hazard ratio (HR) values were calculated using a log-rank test. (**K**) Schematic representation of the protein–protein interaction network for molecules such as *ITGB1*, *ITGB3*, *ITGB5*, *ITGA1*, *ITGA2*, *ITGAV*, *CD44*, and *RHAMM.* Each node represents an individual protein, and different line colors indicate interaction types: red indicates proteins involved in collagen interactions, and blue indicates those involved in fibronectin interactions.

Kaplan–Meier analysis showed that *ITGB1* was not significantly associated with patient outcome in either Luminal A or TNBC cohorts (Figure 2A,F). Similarly, *ITGAV* showed no significant prognostic association in Luminal A or TNBC patients (Figure 2B,G). *ITGB3* also showed no statistically significant association with outcome in either subtype, although a tendency toward higher risk was observed in TNBC patients (Figure 2C,H).

Among the HA-associated genes, *CD44* showed a significant association with improved outcome in Luminal A breast cancer patients, as indicated by a hazard ratio below 1 and a significant log-rank *p*-value (HR = 0.64, log-rank *p* = 0.0042; Figure 2D). However, *CD44* was not significantly associated with patient outcome in the TNBC cohort (HR = 0.84, log-rank *p* = 0.25; Figure 2I).

In contrast, *RHAMM* showed an opposite prognostic pattern in Luminal A breast cancer. High *RHAMM* expression was significantly associated with poorer outcome, as indicated by an increased hazard ratio and a significant log-rank *p*-value (HR = 1.61, log-rank *p* = 0.005; Figure 2E). No significant association was observed in the TNBC cohort (HR = 0.96, log-rank *p* = 0.81; Figure 2J)

STRING network analysis further supported the functional connection between HA-associated receptors and integrin-mediated ECM sensing (Figure 2K). CD44 was associated with collagen-related matrix interactions, while *ITGA1*, *ITGA2,* and *ITGB1* were linked to collagen binding. In addition, *ITGAV*, *ITGB3* and *ITGB1* were associated with fibronectin-binding interactions. These network associations support the selection of these genes in relation to the Col I- and FN-based ECM conditions used in the functional assays.

Overall, the bioinformatic analysis showed that *CD44* and *RHAMM* display subtype-specific prognostic associations in Luminal A breast cancer, whereas *ITGB1*, *ITGAV,* and *ITGB3* were not directly associated with patient outcome in the analyzed cohorts. However, STRING analysis highlighted the functional relationship of HA-associated receptors and integrin subunits with collagen- and fibronectin-related matrix interactions. Thus, the survival analysis provides complementary clinical context rather than direct validation of the in vitro 3D phenotypes. In particular, the absence of significant RFS associations for *ITGB1*, *ITGAV* and *ITGB3* in TNBC indicates that the context-dependent transcriptional changes observed in the experimental models should not be directly extrapolated to patient prognosis.

### 3.3. HA/sHA-Dependent Modulation of 3D Breast Cancer Spheroid Phenotypes

#### 3.3.1. Effect of HA and sHA on Spheroid Growth

The effect of ~50 kDa HA and sHA on spheroid growth was first evaluated in matrix-free 3D cultures of shERβ MDA-MB-231 and Hs578T cells. Spheroid area was monitored over time, together with representative phase-contrast imaging, to assess whether HA-derived molecules altered spheroid growth dynamics.

**Figure 3:**
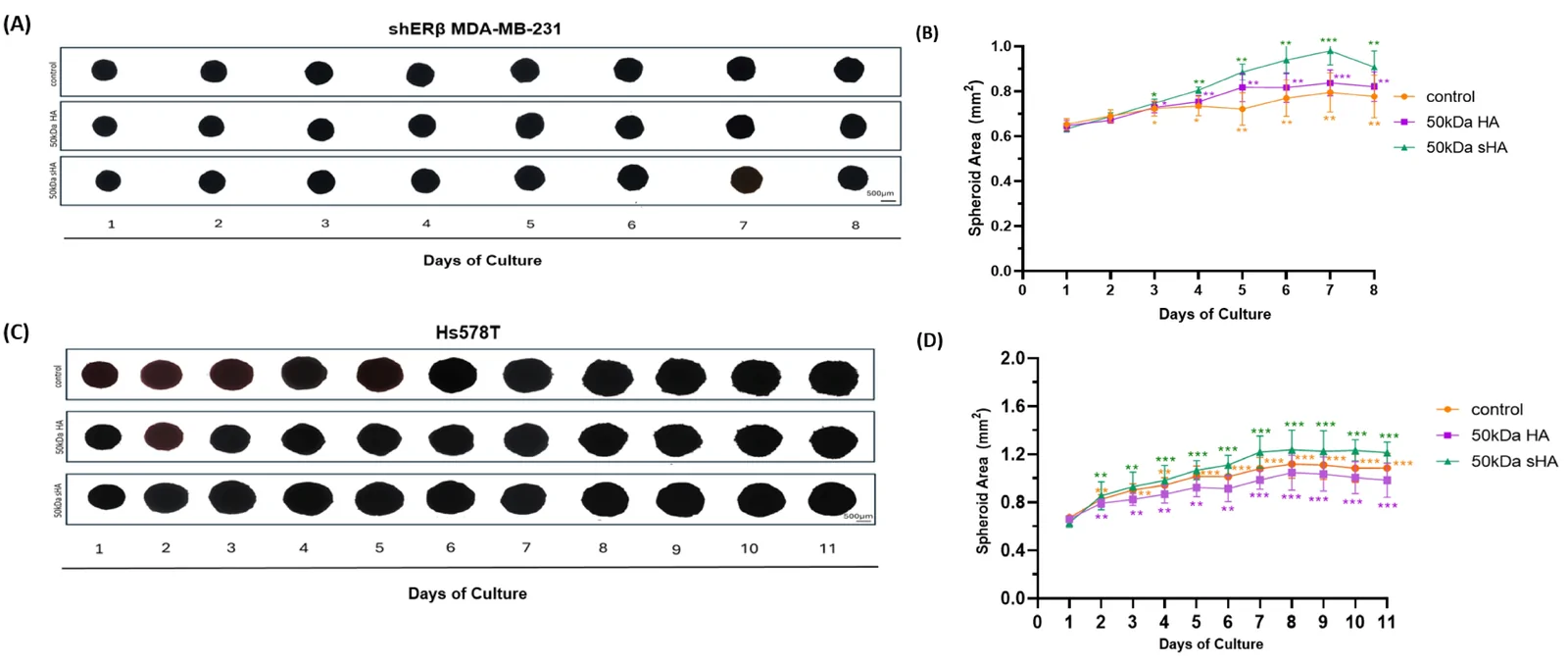
Effects of HA/sHA (~50 kDa) on breast cancer cells’ growth. (**A**) Representative images of the growth rate of shERβ MDA-MB-231 after 24 h of treatment for 8 days (scale bar, 500 μM, magnification 10×). (**B**) The quantification graph of shERβ MDA-MB-231 growth in the presence of HA at a final concentration of 200 μg/mL. (**C**) Representative images of the growth rate of Hs578T after 24 h treatment (scale bar, 500 μM) for 11 days. (**D**) Quantification graph of Hs578T in the presence of HA at a final concentration of 200 μg/mL. Spheroid area was quantified as described in Section 2.12. Data are presented as mean ± SD from triplicate experiments (*n* = 3). Statistical analysis was performed as described in Section 2.12. An asterisk (*) indicates statistically significant differences (*p* < 0.05), two asterisks indicate statistically significant differences *p* < 0.01, and three asterisks indicate statistically significant differences (*p* < 0.001), compared to day 0.

In shERβ MDA-MB-231 cells, control spheroids showed a gradual increase in area during culture. HA-treated spheroids followed a generally similar pattern, with a moderate increase compared with control at later time points (Figure 3B). In contrast, sHA-treated spheroids displayed a more pronounced increase in spheroid area, particularly from the intermediate stages onward, reaching higher values than both control and HA-treated spheroids (Figure 3B). Representative images showed that spheroids remained compact in all conditions (Figure 3A).

In Hs578T cells, control spheroids also showed progressive growth over time (Figure 3C,D). HA-treated spheroids formed compact structures but displayed a lower growth profile compared with control, especially at later time points. sHA-treated spheroids showed increased spheroid area during early and intermediate culture stages, followed by a plateau or slight reduction at later stages (Figure 3D). This suggests that sHA modulated Hs578T spheroid growth in a time-dependent manner.

Overall, HA and sHA differentially affected spheroid growth depending on the breast cancer cell model. In shERβ MDA-MB-231 cells, sHA enhanced spheroid area over time, whereas in Hs578T cells, sHA induced a transient increase followed by stabilization of spheroid growth. These findings indicate that HA-derived molecules regulate the matrix-free 3D spheroid growth dynamics in a cell-line-dependent manner.

#### 3.3.2. HA and sHA Modulate Matrix-Free Spheroid Dissemination

The effect of HA and sHA on spheroid dissemination was next evaluated under matrix-free conditions to assess the intrinsic spreading capacity of pre-formed spheroids in the absence of exogenous ECM substrates.

**Figure 4:**
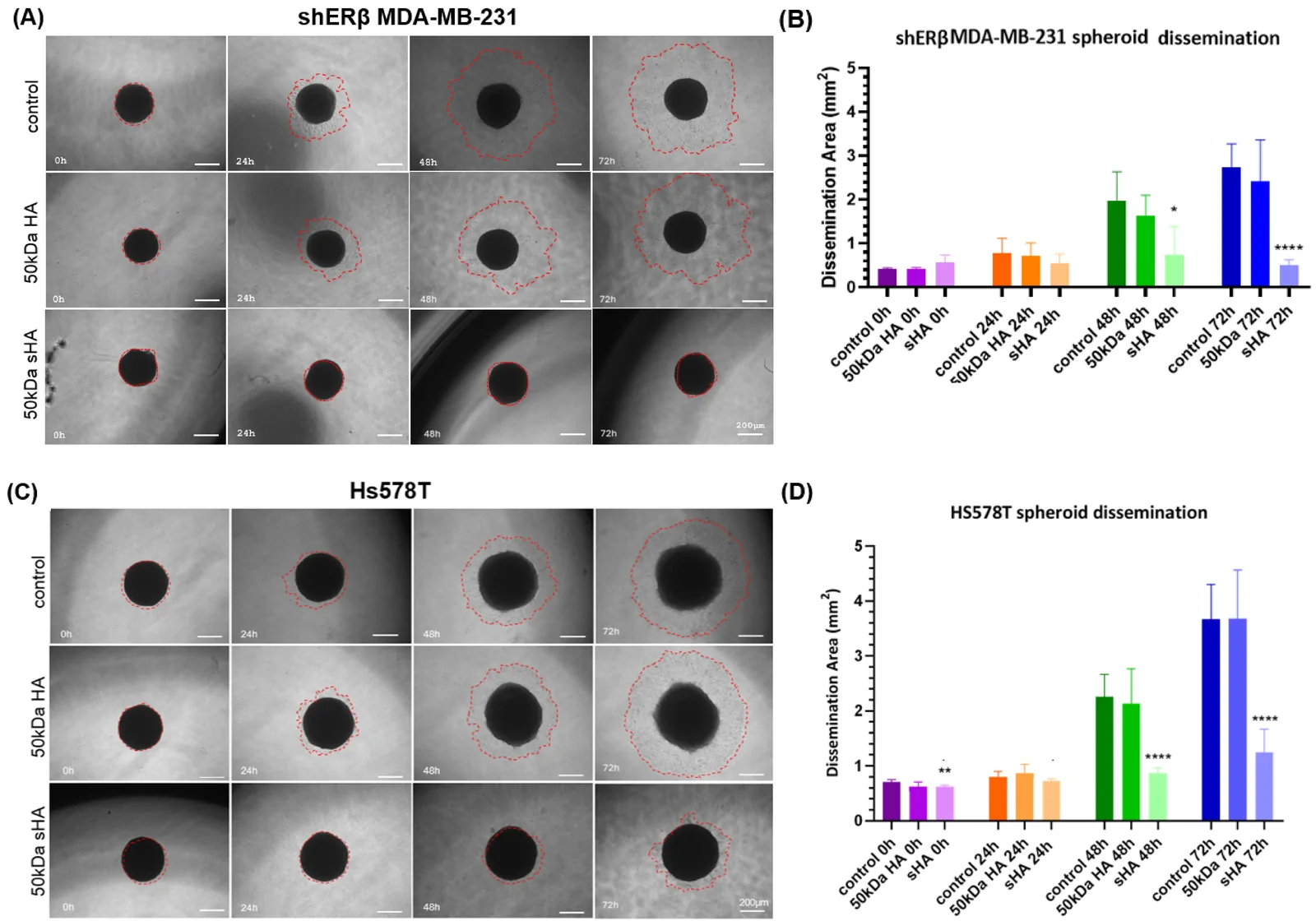
Effect of HA and sHA on spheroid dissemination under matrix-free conditions in shERβ MDA-MB-231 and Hs578T spheroids. (**A**) Representative images from the phase-contrast microscope at 0, 24, 48, and 72 h of dissemination (scale bar, 200 μM, magnification 4×) for shERβ MDA-MB-231. (**B**) Quantification graphs of the spheroid dissemination of shERβ MDA-MB-231 following transfer into a 96-well plate. (**C**) Representative images from the phase-contrast microscope at 0, 24, 48, and 72 h of dissemination (scale bar, 500 μM, magnification 4×) of Hs578T-derived spheroids. (**D**) Quantification graphs of the spheroid dissemination of Hs578T following transfer into a 96-well plate. The red dotted line indicates the boundary of the spheroid dissemination area used for quantitative analysis. Spheroid area was quantified as described in Section 2.12. Data are presented as mean ± SD from triplicate experiments (*n* = 3). Statistical analysis was performed as described in Section 2.12. An asterisk (*) indicates statistically significant differences (*p* < 0.05), two asterisks indicate statistically significant differences *p* < 0.01, and four asterisks indicate statistically significant differences (*p* < 0.0001), compared to the control samples.

In both shERβ MDA-MB-231 and Hs578T cells, control spheroids showed a gradual increase in dissemination area over time, indicating progressive outward spreading from the spheroid core (Figure 4A–D). HA-treated spheroids also maintained dissemination capacity, suggesting that non-sulfated HA did not strongly impair cell spreading under matrix-free conditions (Figure 4A–D).

In contrast, sHA-treated spheroids displayed limited dissemination in both breast cancer cell lines. The outward expansion from the spheroid core was reduced compared with the corresponding control conditions, indicating that sHA interfered with spheroid-derived cell spreading (Figure 4A–D).

Overall, these results suggest that sHA restricts matrix-free spheroid dissemination in both shERβ MDA-MB-231 and Hs578T cells. This supports the idea that sHA not only affects spheroid growth but also modulates the ability of spheroid-derived cells to spread outward from an organized 3D structure.

### 3.4. Spheroid Growth in Matrix-Based Conditions

Matrix-based spheroid growth was evaluated to determine how defined ECM components could influence 3D organization when present from the beginning of spheroid formation. shERβ MDA-MB-231 and Hs578T cells were cultured under matrix-free conditions or within Col I, FN, and mixed Col I/FN environments, and spheroid area and morphology were monitored over time.

**Figure 5:**
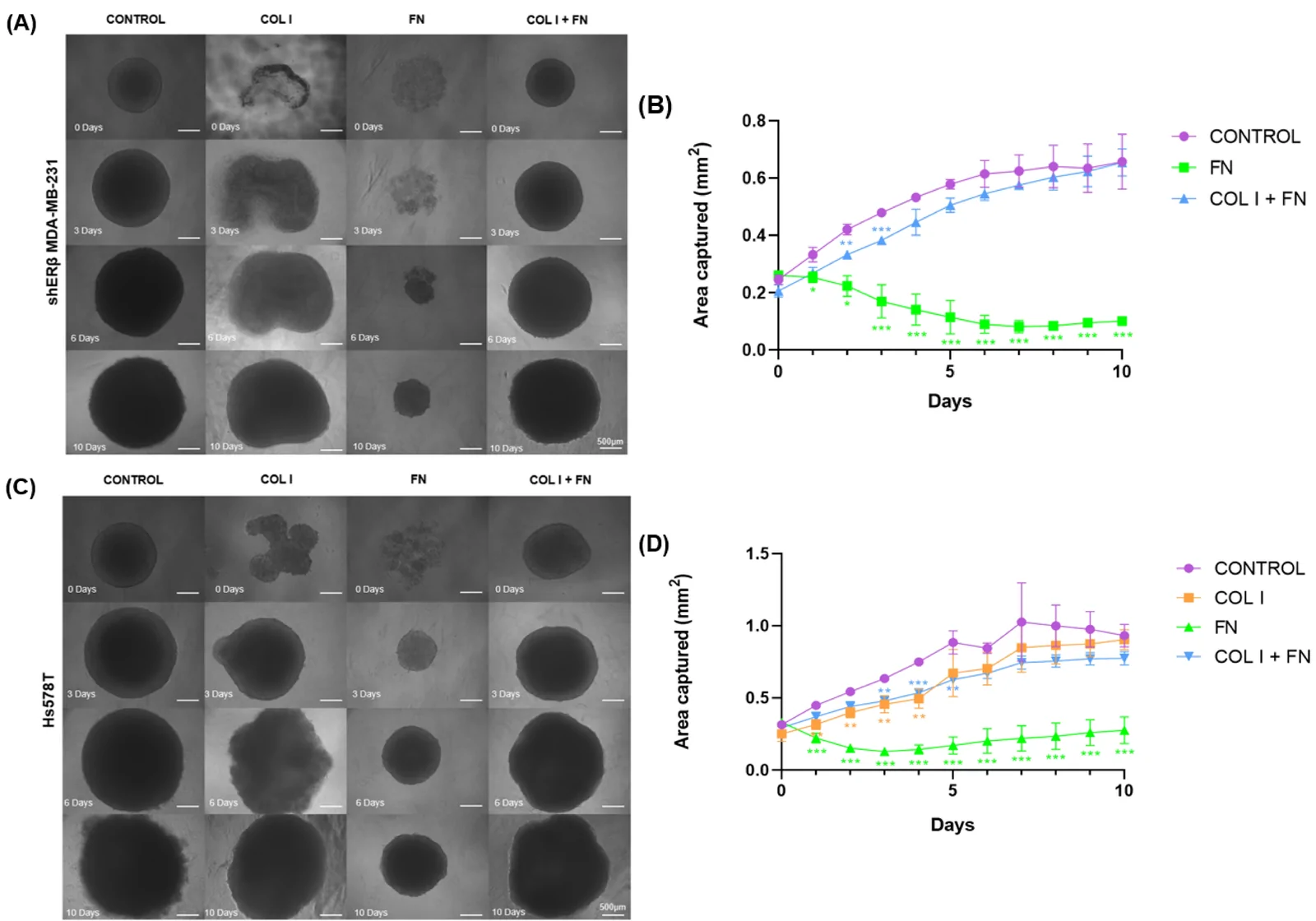
Matrix-dependent spheroid growth of shERβ MDA-MB-231 and Hs578T cells. (**A**) Representative phase-contrast images of spheroids grown under matrix-free, FN, and Col I with FN matrix conditions (scale bar, 500 μM, magnification 10×) of shERβ MDA-MB-231. (**B**) The quantification of the area captured by shERβ MDA-MB-231 cells during spheroid formation, fibronectin and collagen were at final concentrations of 5 μg/mL and 2 mg/mL, respectively. (**C**) Representative phase-contrast images of Hs578T-derived spheroids grown under matrix-free, FN, and Col I with FN matrix conditions (scale bar, 500 μM, magnification 10×). (**D**) The quantification of the area captured by Hs578T cells during spheroid formation, fibronectin and collagen were at final concentrations of 5 μg/mL and 2 mg/mL, respectively. Spheroid area was quantified as described in Section 2.12. Data are presented as mean ± SD from triplicate experiments (*n* = 3). Statistical analysis was performed as described in Section 2.12. An asterisk (*) indicates statistically significant differences (*p* < 0.05), two asterisks indicate statistically significant differences *p* < 0.01, and three asterisks indicate statistically significant differences (*p* < 0.001), compared to the control samples.

In shERβ MDA-MB-231 cells, matrix-free control spheroids formed compact structures and showed a progressive growth pattern. In the presence of FN, spheroids appeared smaller and more compact, suggesting that FN favored a constrained 3D organization. The mixed Col I/FN condition also supported spheroid formation and followed a growth tendency close to the control condition, although the presence of FN appeared to contribute to a more compact architecture. Col I produced a more variable spheroid phenotype, indicating that collagen-rich environments may provide less uniform structural cues during early spheroid assembly (Figure 5A,B).

In Hs578T cells, control spheroids showed a progressive increase in area over time. FN-containing conditions generated smaller and more compact spheroids compared with the control, suggesting restricted spheroid expansion. Col I produced relatively smaller but looser spheroid structures, indicating that collagen-rich matrices affected both spheroid size and organization. The mixed Col I/FN condition followed a similar tendency to Col I at early stages; however, over time, spheroid size became more reduced compared with collagen alone, probably due to the presence of fibronectin (Figure 5C,D).

Overall, these findings show that ECM composition strongly influences matrix-based spheroid growth and architecture in both breast cancer models. FN-containing environments favored smaller and more compact spheroid organization, whereas Col I produced a looser and more matrix-dependent phenotype, particularly in Hs578T cells. Quantitative area analysis was restricted to conditions in which a single continuous spheroid-like structure could be reliably delineated.

### 3.5. The Dissemination of shERβ MDA-MB-231 and Hs578T Spheroids Under Matrix-Free and Matrix-Based Conditions

Matrix-based dissemination assays were performed to evaluate how defined ECM substrates influence the outward spreading of pre-formed breast cancer spheroids. In contrast to matrix-based spheroid growth, where ECM components were present from the beginning of spheroid formation, this assay examined the dissemination capacity of already established spheroids after transfer onto matrix-free or matrix-coated surfaces.

**Figure 6:**
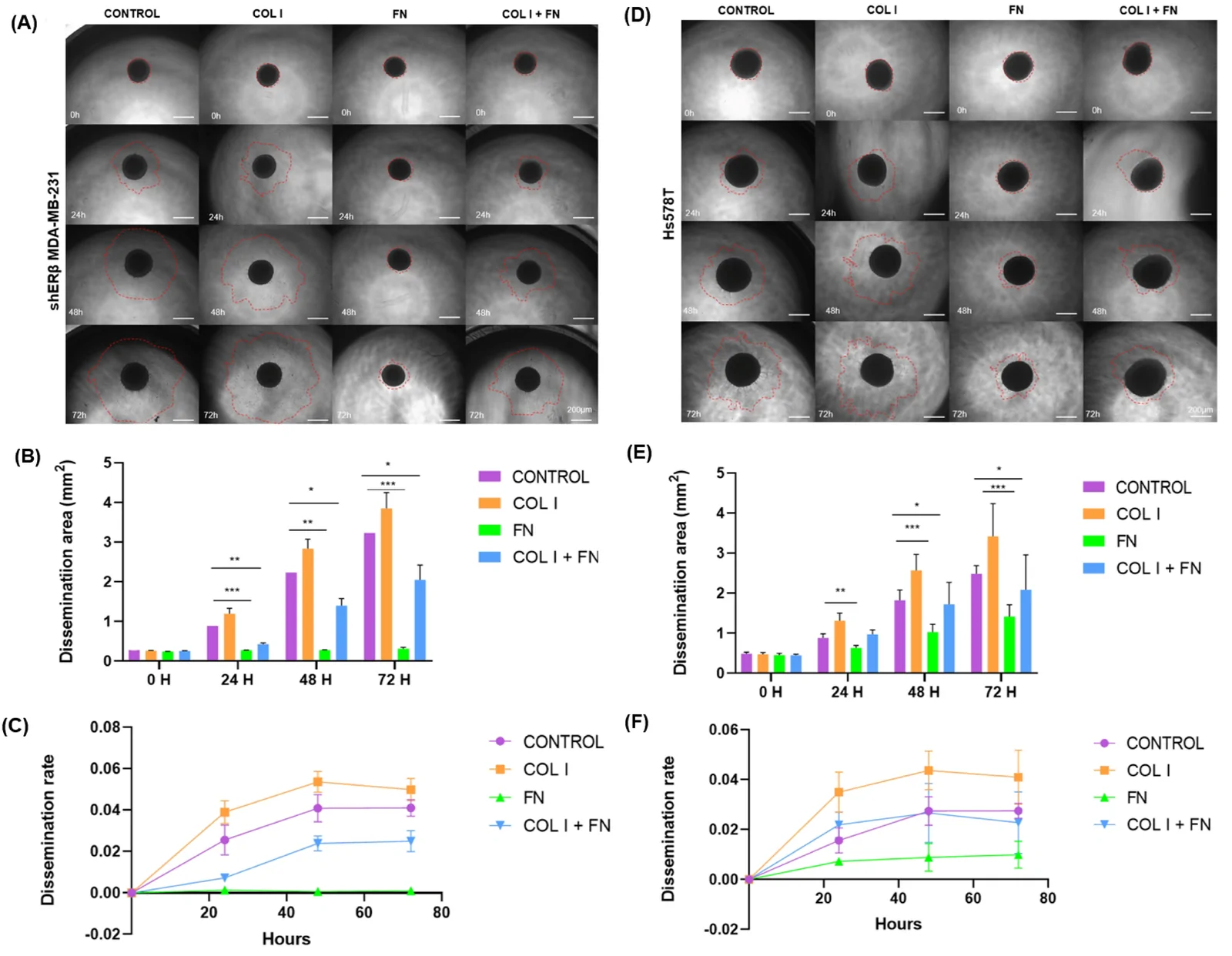
The effect of matrix context on shERβ MDA-MB-231 and Hs578T spheroid dissemination. Graphs (**A**–**C**) refer to shERβ MDA-MB-231-derived spheroids, while graphs (**D**–**F**) refer to Hs578T-derived spheroids. (**A**) Representative images from the phase-contrast microscope at 0, 24, 48, and 72 h of dissemination (scale bar, 200 μM, magnification 4×). (**B**) Quantification graphs of the spheroid dissemination following transfer onto matrix-free or matrix-based coating. (**C**) Graphical representation of the dissemination rate (Δdissemination/Δt) for each condition. (**D**) Representative images from the phase-contrast microscope at 0, 24, 48, and 72 h of dissemination (scale bar, 500 μM, magnification 4×). (**E**) Quantification graphs of the spheroid dissemination following transfer onto matrix-free or matrix-based coating. The red dotted line indicates the boundary of the spheroid dissemination area used for quantitative analysis. Graphical representation of the dissemination rate (Δdissemination/Δt) for each condition. Spheroid area was quantified as described in Section 2.12. Data are presented as mean ± SD from triplicate experiments (*n* = 3). Statistical analysis was performed as described in Section 2.12. An asterisk (*) indicates statistically significant differences (*p* < 0.05), two asterisks indicate statistically significant differences *p* < 0.01, and three asterisks indicate statistically significant differences (*p* < 0.001), compared to the control samples.

In shERβ MDA-MB-231 cells, matrix-free control spheroids showed a gradual increase in dissemination area over time, reflecting their baseline spreading capacity. Col I produced the strongest dissemination response, with cells expanding extensively from the spheroid core, especially at later time points (Figure 6A,B). This was also supported by the dissemination-rate analysis, which showed a stronger spreading tendency in Col I compared with the other matrix conditions (Figure 6C). The mixed Col I/FN condition also supported dissemination, but to a lower extent than Col I alone, indicating that fibronectin modified the collagen-driven spreading response. In contrast, fibronectin alone showed the weakest dissemination pattern, with limited outward expansion from the spheroid core (Figure 6A,B).

A similar ECM-dependent pattern was observed in Hs578T cells. Control spheroids showed progressive outward spreading under matrix-free conditions, while Col I supported a clear dissemination response. FN-containing conditions restricted spheroid spreading compared with Col I, suggesting that FN may favor local adhesion or constrained organization rather than extensive outward movement. The mixed collagen/fibronectin condition produced an intermediate response, indicating that fibronectin can limit or reshape collagen-driven dissemination (Figure 6D–F).

Overall, Col I provided the most permissive matrix context for spheroid dissemination in both breast cancer models, whereas FN-containing environments restricted outward spreading. These findings demonstrate that ECM composition regulates not only spheroid growth and organization but also the ability of spheroid-derived cells to disseminate from an established 3D structure.

### 3.6. Matrix-Based Spheroid Growth Following HA/sHA Integration

To further examine the effect of HA-derived molecules within defined ECM environments, matrix-based spheroid growth was evaluated after incorporation of ~50 kDa HA or sHA into Col I-, FN-, and collagen/fibronectin-containing matrices. Spheroid formation and organization were monitored over time by phase-contrast microscopy, and spheroid area was quantified when a single measurable spheroid-like structure was formed.

**Figure 7:**
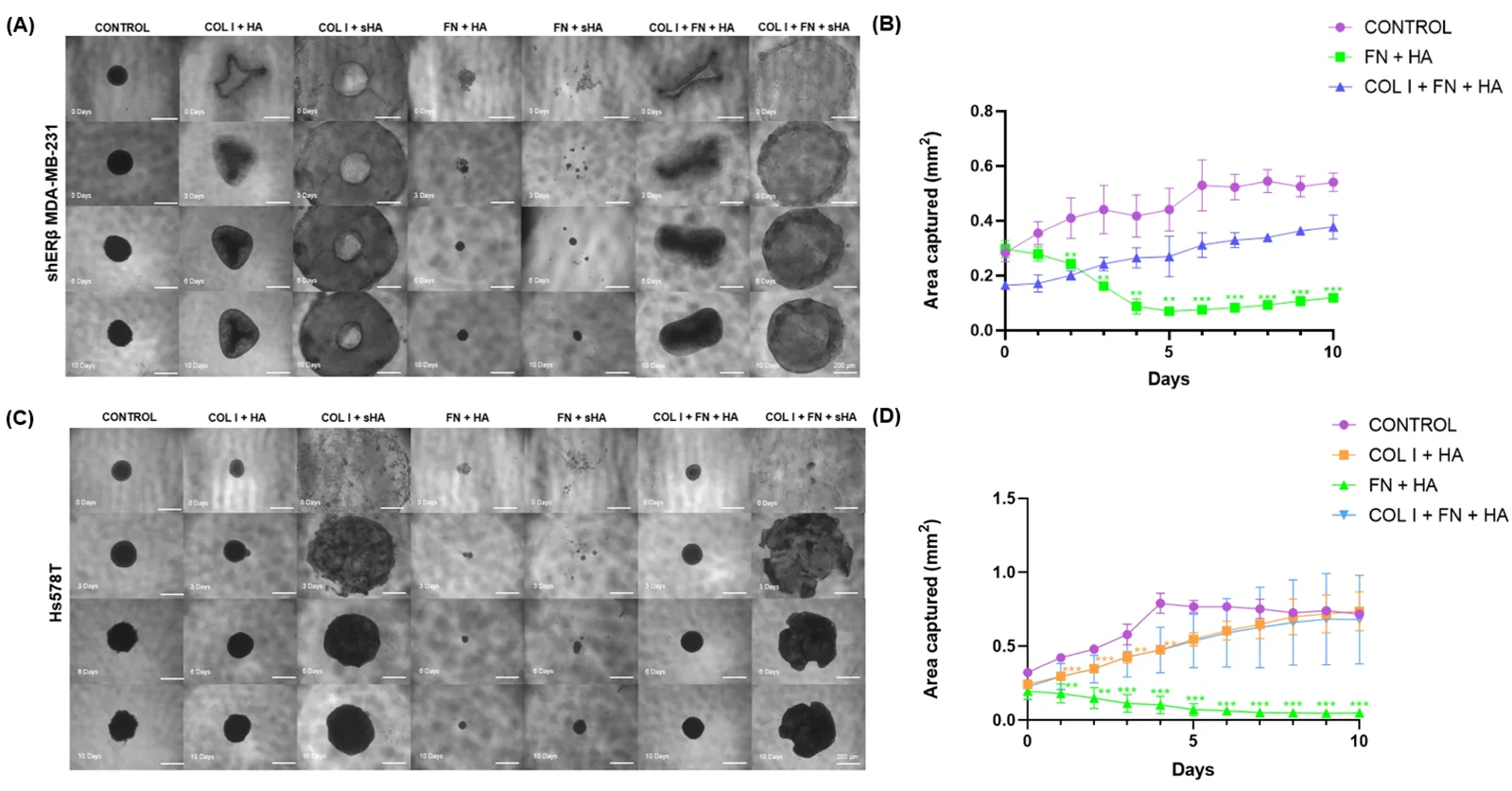
Matrix-dependent spheroid growth of shERβ MDA-MB-231 and Hs578T cells following incorporation of HA/sHA. Graphs (**A**,**B**) refer to shERβ MDA-MB-231 cells, while graphs (**C**,**D**) refer to Hs578T cells. (**A**) Representative phase-contrast images of spheroids grown under distinct matrix conditions (scale bar, 200 μM, magnification 4×). (**B**) The quantification of the area captured by the cells during spheroid formation, fibronectin (FN), and collagen type I (Col I) was at final concentrations of 5 μg/mL and 2 mg/mL, respectively, while the HA concentration was 200 μg/mL. (**C**) Representative phase-contrast images of spheroids grown under distinct matrix conditions (scale bar, 500 μM, magnification 4×). (**D**) The quantification of the area captured by the cells during spheroid formation, fibronectin, and collagen was at final concentrations of 5 μg/mL and 2 mg/mL, respectively, while the HA concentration was 200 μg/mL. Spheroid area was quantified as described in Section 2.12. Data are presented as mean ± SD from triplicate experiments (*n* = 3). Statistical analysis was performed as described in Section 2.12. Two asterisks indicate statistically significant differences *p* < 0.01, and three asterisks indicate statistically significant differences (*p* < 0.001), compared to the control samples.

In shERβ MDA-MB-231 cells, incorporation of non-sulfated HA generally maintained the trends observed in the corresponding matrix-only conditions. Fibronectin-containing matrices supported the formation of smaller and more compact structures, whereas mixed collagen/fibronectin matrices preserved a matrix-dependent growth pattern. However, Col I supplemented with HA again showed high variability in spheroid formation and was therefore not included in the quantitative analysis (Figure 7A,B).

In contrast, sHA-containing matrices produced a more pronounced alteration of 3D organization. Under these conditions, shERβ MDA-MB-231 cells failed to form typical compact spheroids, regardless of the ECM context. Instead, cells formed diffuse cell masses or distinct cell spots within the matrix substrate. Because these conditions did not generate a single continuous spheroid area, sHA-containing matrices were evaluated qualitatively and were not included in area quantification (Figure 7A). Instead, their morphology was characterized by diffuse cell masses, loose aggregates, multiple discrete colonies, or spatially separated cell areas, depending on the matrix context.

A similar pattern was observed in Hs578T cells. Incorporation of non-sulfated HA generally preserved the matrix-dependent behavior observed in the absence of HA, with spheroid morphology remaining dependent on the surrounding ECM composition. However, sHA strongly affected early spheroid assembly. At early stages, Hs578T cells failed to organize into compact spheroids, particularly within ECM-rich conditions. Instead, cells formed looser structures in collagen-containing matrices and smaller cell colonies or distinct cell areas in fibronectin-containing environments (Figure 7C,D).

In sHA-containing conditions, distinct cell areas became evident after the first days of culture, particularly from approximately day 3 onward. For this reason, these conditions were not quantified as conventional spheroid areas, since the observed structures did not correspond to a single organized spheroid. Accordingly, area-based comparisons were restricted to conditions in which a continuous spheroid boundary could be reliably defined. Over time, some structures became more defined, but they remained less compact and less uniformly organized than spheroids formed under HA- or matrix-only conditions (Figure 7D).

Overall, incorporation of non-sulfated HA largely maintained matrix-dependent spheroid growth patterns, whereas sHA disrupted organized spheroid formation in both breast cancer models. These findings suggest that sHA affects not only spheroid size, but also the ability of breast cancer cells to establish coordinated 3D architecture within ECM-rich microenvironments.

### 3.7. HA/sHA-Dependent Modulation of Matrix-Based Spheroid Dissemination in Hs578T Cells

**Figure 8:**
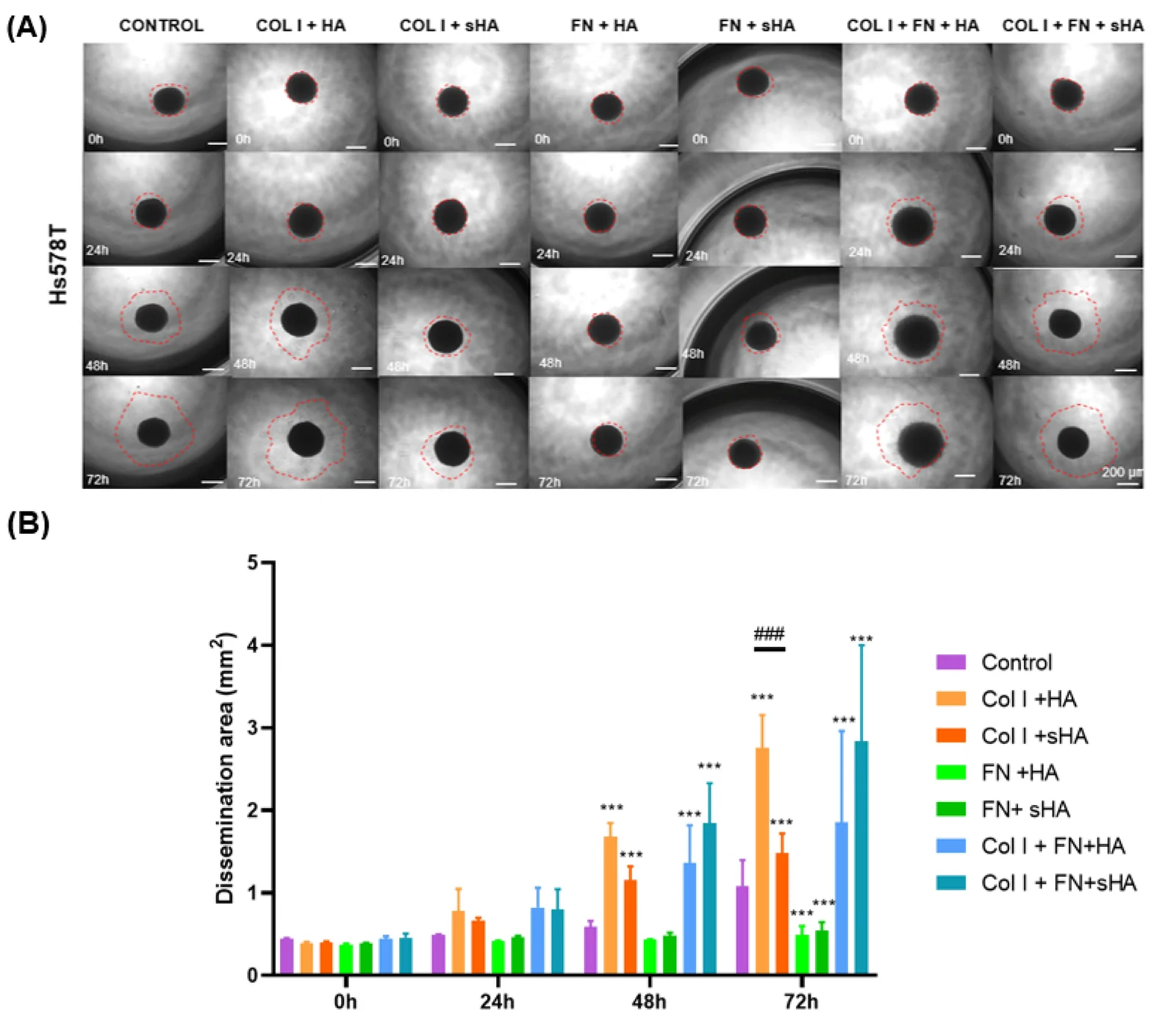
HA/sHA incorporation within ECM scaffolds modulates matrix-based dissemination of Hs578T spheroids. (**A**) Representative images from the phase-contrast microscope at 0, 24, 48, and 72 h of dissemination (scale bar, 200 μM, magnification 4×). (**B**) Quantification graphs of the spheroid dissemination following transfer onto matrix-free or matrix-based coating. The red dotted line indicates the boundary of the spheroid dissemination area used for quantitative analysis. Spheroid area was quantified as described in Section 2.12. Data are presented as mean ± SD from triplicate experiments (*n* = 3). Statistical analysis was performed as described in Section 2.12. Three asterisks indicate statistically significant differences (*p* < 0.001), compared to the control samples. Statistically significant differences between HA- and sHA-treated groups within the same matrix condition and at the same time point are indicated by hashes. Statistically significant differences (*p* < 0.001) are indicated by three hashes (###).

**Figure 9:**
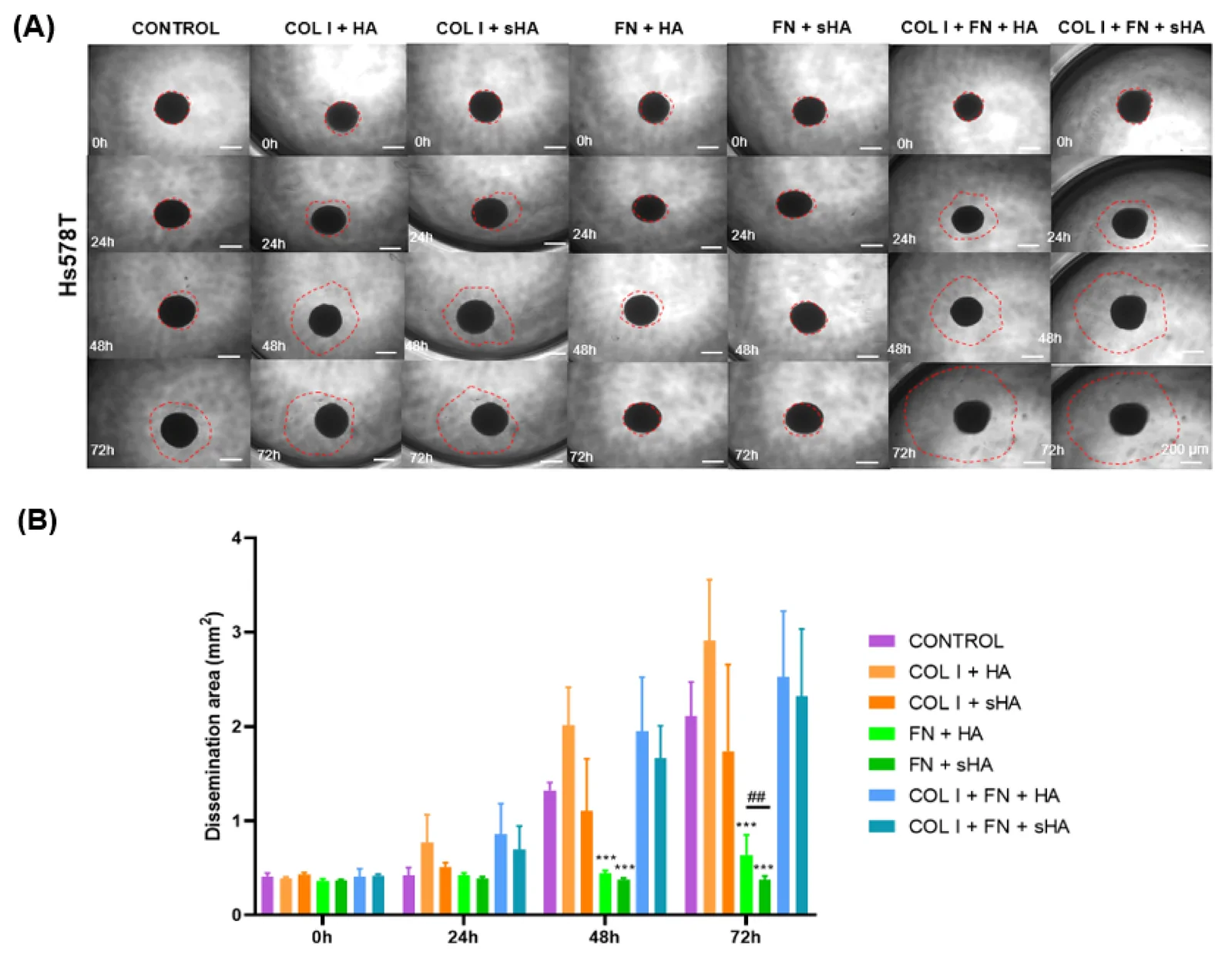
HA/sHA-preconditioned Hs578T spheroids display matrix-dependent dissemination responses. (**A**) Representative images from the phase-contrast microscope at 0, 24, 48, and 72 h of dissemination (scale bar, 200 μM, magnification 4×). (**B**) Quantification graphs of the spheroid dissemination following transfer onto matrix-free or matrix-based coating. The red dotted line indicates the boundary of the spheroid dissemination area used for quantitative analysis. Spheroid area was quantified as described in Section 2.12. Data are presented as mean ± SD from triplicate experiments (*n* = 3). Statistical analysis was performed as described in Section 2.12. Three asterisks indicate statistically significant differences (*p* < 0.001), compared to the control samples. Statistically significant differences between HA- and sHA-treated groups within the same matrix condition and at the same time point are indicated by hashes. Statistically significant differences (*p* < 0.01) are indicated by two hashes (##).

Based on the reproducible matrix-dependent dissemination profile of Hs578T spheroids and their clear response to sHA during ECM-rich spheroid organization, the final dissemination assays were focused on this cell model. This allowed a more targeted evaluation of how HA and sHA influence matrix-dependent spreading without the additional variability observed in some matrix-based spheroid formation conditions. Accordingly, the findings presented in Figure 8 and Figure 9 should be interpreted as specific to the Hs578T model.

#### 3.7.1. Matrix-Based Dissemination Following HA/sHA Incorporation Within the ECM Scaffold

To evaluate whether HA-derived molecules directly modify the permissiveness of the surrounding matrix, HA or sHA was incorporated within the ECM scaffold before spheroid transfer. Pre-formed Hs578T spheroids were then placed onto Col I-, FN- or mixed collagen/fibronectin-containing matrices, with or without HA or sHA incorporation, and dissemination was monitored over time.

Consistent with the matrix-based dissemination assay without hyaluronan derivatives, Hs578T spheroids showed a progressive increase in dissemination area over time. Col I remained the most permissive matrix condition, supporting stronger outward expansion from the spheroid core. The mixed collagen/fibronectin condition produced an intermediate phenotype, whereas fibronectin showed a more restrictive effect, particularly at later time points (Figure 8A,B).

Interestingly, incorporation of sHA within the Col I scaffold restricted spheroid dissemination at later time points. This effect became evident after 48 h and was more pronounced at 72 h, with dissemination in the collagen/sHA condition reduced compared with the collagen alone and collagen/HA conditions. Therefore, while Col I normally provides a permissive substrate for Hs578T spheroid spreading, sHA incorporation within this matrix reduced its dissemination-supportive effect (Figure 8A,B).

Overall, these findings suggest that sHA can modify the functional properties of collagen-rich ECM scaffolds and limit collagen-driven spheroid dissemination. This indicates that the anti-disseminative effect of sHA may depend not only on direct effects on cancer cells but also on its incorporation into the surrounding ECM.

#### 3.7.2. Matrix-Based Dissemination of HA/sHA-Preconditioned Spheroids

To further distinguish scaffold-associated effects from direct effects on spheroids, Hs578T spheroids were preconditioned with HA or sHA before being transferred onto defined ECM substrates. HA or sHA treatment was repeated every 48 h, following the treatment scheme used in previous in vivo experimental approaches, in order to mimic repeated exposure to HA-derived molecules.

Overall, HA/sHA-preconditioned spheroids maintained the general ECM-dependent dissemination pattern observed in the matrix-based spreading assay without hyaluronan derivatives. Col I supported the strongest outward spreading, the mixed collagen/fibronectin condition produced an intermediate response, and FN remained the most restrictive matrix context (Figure 9A,B).

However, a distinct effect was observed at later time points in FN-containing conditions. sHA-preconditioned spheroids showed further restriction of dissemination on fibronectin compared with the corresponding untreated or HA-treated conditions. This differs from the previous experiment, where the strongest sHA-associated restriction was observed when sHA was incorporated within the Col I scaffold (Figure 9A,B).

These results suggest that the mode of sHA exposure influences its effect on spheroid dissemination. When sHA is incorporated into the matrix, it mainly reduces collagen-driven dissemination. In contrast, when spheroids are preconditioned with sHA, the strongest restriction appears in the fibronectin context. Together, these findings indicate that sHA can modulate matrix-dependent dissemination through both scaffold-associated and spheroid-associated mechanisms.

## 4. Discussion

Breast cancer progression is strongly influenced by the interaction between tumor cells and the extracellular matrix. In this study, defined ECM substrates, particularly Col I, FN, and their combination, treated with or without HA and sHA, were used to investigate how they could regulate breast cancer spheroid behavior and matrix-associated receptor programs. By combining matrix-free 2D cultures and 3D spheroids as well as matrix-based 3D platforms, functional assays and gene expression analysis were performed. This work provides evidence that breast cancer cell behavior is shaped by ECM composition, HA sulfation, and model-dependent matrix-sensing responses.

The first major finding of this study is that HA and sHA differentially regulate gene expression programs associated with matrix sensing and HA-related signaling. In shERβ MDA-MB-231 cells, HA- and sHA-treated conditions induced broad differential expression of adhesion-related integrins. Several integrin subunits, including *ITGA1*, *ITGB3*, and *ITGB5*, were upregulated across different culture conditions, while *ITGAV* and *ITGB1* displayed more context-dependent regulation. Notably, sHA induced *ITGB1* expression in 3D spheroids, suggesting that sulfation of the HA may promote a specific adaptation of β1-associated matrix-sensing programs under 3D conditions. In contrast, Hs578T cells showed a stronger response in a 3D setting, with downregulation of several collagen- and fibronectin-associated integrins in 3D spheroids and persistent induction of *ITGB3*. These differences indicate that the two breast cancer models respond to HA-derived molecules and 3D organization through distinct receptor-remodeling programs.

The HA-associated receptor profile further supported this model-dependent regulation. In shERβ MDA-MB-231 cells, sHA reduced *RHAMM* expression in both 2D and 3D conditions, while *CD44* was increased by sHA in 2D and by the transition to 3D culture. This opposite regulation of *RHAMM* and *CD44* suggests that sHA may shift HA-associated signaling toward a different receptor balance. In Hs578T cells, *CD44* showed only limited modulation, whereas *RHAMM* was reduced by sHA in 2D and further downregulated in 3D spheroids. Since *RHAMM* is closely related to HA-mediated motility and cytoskeletal remodeling, its downregulation may be relevant to the restricted dissemination observed under sHA-treated conditions.

Bioinformatic analysis provided complementary clinical and functional context for the selected matrix- and HA-associated genes. *RHAMM* and *CD44* showed subtype-specific prognostic associations in Luminal A breast cancer, with high *RHAMM* expression associated with poorer outcome and high *CD44* expression associated with improved outcome. In contrast, the analyzed integrins were not significantly associated with patient outcome in the selected TNBC cohort. These survival data should not be interpreted as direct validation of the specific 3D phenotypes observed in shERβ MDA-MB-231 and Hs578T cells. Rather, they indicate that the clinical relevance of these genes may depend on breast cancer subtype and may differ from their context-dependent role in ECM sensing within experimental 3D models. Additional exploratory Kaplan–Meier analyses of overall survival suggested that high ITGB1 expression was associated with poorer prognosis in TNBC patients (HR = 3.26, *p* = 0.015), whereas *ITGAV* and *ITGB3* showed no significant associations. No significant associations were observed for these integrins in the ER+/PR+ cohort. However, these analyses were not included in the main results due to the relatively limited number of patients, particularly in the TNBC cohort, and the associated uncertainty in the survival estimates. Therefore, these observations should be interpreted cautiously and require validation in larger, independent patient cohorts. STRING analysis connected *CD44* and selected integrin subunits with collagen- and fibronectin-associated interactions, supporting the biological rationale for examining these molecules in the Col I- and FN-based experimental conditions. However, STRING associations may represent direct or indirect functional relationships derived from experimental evidence, database annotations, co-expression, text mining, or computational prediction and therefore do not demonstrate that these interactions are active or causal in the present models. Accordingly, the bioinformatic findings should be interpreted as supportive and hypothesis-generating rather than definitive mechanistic evidence.

At the functional level, HA and sHA altered breast cancer spheroid growth in a cell-line-dependent manner. In matrix-free conditions, shERβ MDA-MB-231 spheroids remained compact, with sHA increasing spheroid area over time. In Hs578T cells, sHA induced an early and intermediate increase in spheroid area followed by stabilization at later time points. These findings indicate that sHA does not simply block spheroid formation. Instead, its effect depends on the biological context of each cell model and on the stage of spheroid development. Importantly, in matrix-free dissemination assays, sHA-treated spheroids showed limited outward spreading in both cell lines, suggesting that sHA can restrict the ability of spheroid-derived cells to disseminate from an organized 3D structure.

Defined ECM environments further revealed that collagen type I and fibronectin generate distinct spheroid phenotypes. In matrix-based spheroid growth assays, fibronectin-containing conditions generally favored smaller and more compact spheroid structures, whereas Col I produced a looser and more matrix-dependent phenotype, particularly in Hs578T cells. These observations are consistent with the different biological roles of these ECM proteins. FN provides adhesive cues that may promote local anchorage and compaction, while Col I can create a more permissive fibrillar environment that supports remodeling and outward expansion. The mixed collagen/fibronectin condition produced intermediate or modified phenotypes, indicating that combined ECM environments do not simply reproduce one matrix effect, but create a distinct microenvironmental context.

Matrix-based dissemination assays confirmed that ECM composition regulates the spreading capacity of pre-formed spheroids. Col I was the most permissive substrate for outward dissemination in both breast cancer models, while fibronectin-containing environments restricted spreading. The mixed matrix produced an intermediate phenotype, suggesting that FN can limit or reshape collagen-driven dissemination. These findings highlight the importance of distinguishing spheroid formation from spheroid dissemination. When ECM components are present from the beginning of spheroid assembly, they influence 3D architecture and compaction. When spheroids are transferred onto ECM-coated surfaces, the same matrix components regulate the ability of cells to escape from the spheroid core and spread outward.

The incorporation of HA or sHA within ECM-rich scaffolds added another layer of complexity. Native, non-sulfated HA generally preserved matrix-dependent spheroid organization, whereas sHA disrupted the formation of compact and organized spheroids. In both shERβ MDA-MB-231 and Hs578T cells, sHA-containing matrices led to diffuse cell masses, loose structures, or distinct cell colonies rather than a single measurable spheroid. For this reason, these conditions were not quantified as conventional spheroid areas. This qualitative phenotype is biologically important because it suggests that sHA interferes with coordinated 3D assembly, cell compaction, and cell–matrix communication within matrix substrate-rich environments.

The final dissemination assays in Hs578T cells further indicated that the mode of sHA exposure influences its functional outcome. When sHA was incorporated within the ECM scaffold, the most evident restriction of dissemination occurred in collagen-rich conditions at later time points. This suggests that sHA can modify the functional properties of collagen-containing matrices and reduce their normally permissive effect on spheroid spreading. In contrast, when spheroids were preconditioned with HA or sHA before transfer onto ECM substrates, the strongest additional restriction was observed on FN. These findings suggest that sHA may act through both scaffold-associated and cell-associated mechanisms. In one case, sHA may alter how the matrix supports dissemination; in the other, it may modify how spheroid-derived cells respond to specific ECM cues. These findings are consistent with previous reports describing anti-tumor effects of sHA in breast and other cancer models, while also supporting the concept that its activity is strongly dependent on the tumor and ECM context [27,28,30].

Taken together, the present findings support a model in which ECM composition, HA sulfation, and receptor-mediated matrix sensing jointly regulate breast cancer spheroid behavior. Col I promoted dissemination, FN favored compact organization and restricted spreading, while sHA disrupted organized spheroid formation and limited dissemination depending on the matrix context and mode of exposure. At the molecular level, these functional changes were accompanied by model-dependent remodeling of integrin and HA-associated gene expression. However, the present data establish an association rather than a causal relationship between the transcriptional changes and the observed spheroid phenotypes. These findings therefore suggest that breast cancer cells adapt their matrix-sensing programs according to culture conditions, ECM composition, and HA-derived signals, while the direct contribution of individual receptors or pathways requires functional validation.

This study has some limitations. First, all experiments were performed using in vitro 2D and 3D systems. Although the 3D matrix-based models reproduce selected aspects of tumor–ECM interactions, they do not capture the full cellular, biochemical, mechanical, and spatial complexity of the tumor microenvironment in vivo. Therefore, the observed effects on spheroid organization and dissemination cannot be directly extrapolated to tumor behavior in vivo, and future studies using appropriate in vivo breast cancer models will be required to assess their relevance to tumor growth, invasion and matrix remodeling. Moreover, the concentrations of Col I, FN and HA/sHA used here were selected to establish reproducible and defined experimental matrix environments and should not be interpreted as quantitative reconstructions of their absolute concentrations within human breast tumors, where ECM composition and distribution are highly heterogeneous.

The present study examined HA and sHA at a single molecular-weight range (~50 kDa) and concentration (200 µg/mL). The selected concentration was based on previous experimental work by Koutsakis et al. using comparable HA/sHA treatment conditions in breast cancer cells [30]. Importantly, HA-mediated effects on breast cancer cell functional properties, morphology, and matrix-associated gene expression have been shown to depend on molecular size [22]. Therefore, the responses observed here should be considered specific to the ~50 kDa HA/sHA fragments and the concentration examined, while other molecular weights or concentrations may produce distinct biological effects.

In addition, gene expression analysis was performed at the mRNA level and therefore reflects transcriptional regulation rather than receptor abundance, localization, or activation. Complementary protein-level analyses, including Western blotting, flow cytometry, or immunofluorescence, together with functional inhibition experiments, will be required to determine whether the observed transcriptional changes translate into corresponding alterations at the protein and functional levels. Moreover, spheroid area was used as the principal quantitative endpoint for matrix-based growth assays and could only be applied when a single continuous structure was formed. Complementary measurements of total cell number, metabolic activity or viability together with alternative image-based parameters such as circularity, solidity or dispersal area, would help distinguish effects on cell growth and survival from changes in spheroid compaction and spatial organization.

The final HA/sHA dissemination assays were performed in Hs578T cells, which provided a reproducible model for evaluating scaffold- and spheroid-associated sHA effects, but future studies should extend this comparison to additional breast cancer models. Furthermore, because shERβ MDA-MB-231 represents an ERβ-suppressed derivative of the parental MDA-MB-231 cell line, the responses observed in this model should not be generalized to TNBC as a whole. Validation in parental MDA-MB-231 cells and additional TNBC models will be required to determine the broader applicability of these findings. The bioinformatic analyses also have inherent limitations. Kaplan–Meier Plotter integrates heterogeneous publicly available datasets that may differ in patient composition, treatment, sample processing, and molecular characteristics, while STRING includes both experimentally supported and computationally inferred associations. These analyses therefore provide supportive clinical and functional context but cannot establish direct mechanistic relationships within the experimental models used in this study. Finally, direct measurements of matrix stiffness, receptor activation, and downstream signaling pathways would strengthen the mechanistic interpretation of sHA-mediated effects.

Overall, this work demonstrates that defined ECM contexts are active regulators of breast cancer spheroid architecture, dissemination, and receptor-associated matrix responses. The results support sulfated hyaluronan as a bioactive ECM-associated molecule capable of altering tumor–matrix communication, particularly within collagen- and fibronectin-rich environments. These findings highlight the value of 3D ECM-based breast cancer models for studying matrix-dependent tumor behavior and provide a basis for further investigation of sHA as a potential modulator of tumor-supportive extracellular matrix interactions.

## 5. Conclusions

This study demonstrates that defined ECM contexts actively regulate breast cancer spheroid behavior and matrix-associated receptor programs. Col I, FN, and mixed Col I/FN environments generated distinct 3D phenotypes, affecting spheroid growth, organization, and dissemination. Col I acted as the most permissive matrix for outward spheroid spreading, whereas FN-containing environments favored more compact organization and restricted dissemination.

HA/sHA further modified these matrix-dependent responses. HA generally preserved spheroid-like organization in a matrix-dependent manner, while sHA produced more pronounced effects on 3D architecture. In ECM-rich conditions, sHA disrupted the formation of compact and organized spheroids, leading to loose structures, diffuse cell masses, or smaller cell colonies depending on the matrix context. Moreover, sHA restricted spheroid dissemination under specific conditions, suggesting that its biological activity depends on both the surrounding ECM environment and the mode of exposure.

At the molecular level, HA/sHA treatment and 3D culture dimensionality differentially regulated integrin- and HA-associated gene expression. shERβ MDA-MB-231 cells showed broader HA/sHA-responsive remodeling of adhesion-related integrins, whereas Hs578T cells displayed stronger dimensionality-driven regulation. *CD44* and *RHAMM* also showed distinct cell-line-dependent responses, which were associated with the observed matrix-dependent phenotypes but do not establish a direct causal role for these receptors.

Overall, these findings support a model in which ECM composition, HA sulfation, and receptor-mediated matrix sensing collectively shape breast cancer spheroid architecture and dissemination. The results highlight the value of 3D ECM-based models for studying tumor–matrix communication and support further investigation of sulfated hyaluronan as a bioactive ECM-associated molecule capable of modulating breast cancer cell behavior.

## Figures and Tables

**Table 1:** Real-time PCR primer pair sequences used in this study.

Gene	Primer Sequence (5′-3′)	Annealing T (°C)
*18S rRNA*	F: CAGGUCUGUGAUGCCCUUAGA R: GCUUAUGACCCGCACUUACUG	60
*ITGA1*	F: GGTTCCTACTTTGGCAGTATT R: AACCTTGTCTGATTGAGAGCA	56
*ITGA2*	F: GGAACGGGACTTTCGCAT R: GGTACTTCGGCTTTCTCATCA	58
*ITGAV*	F: AATCTTCCAATTGAGGATATCAC R: AAAACAGCCAGTAGCAACAAT	55
*ITGB1*	F: AAAGGAGAAAAGAAAGACAC R: CGTAAAATAGAACCAACAGT	51
*ITGB3*	F: CCGTGACGAGATTGAGTCA R: AGGATGGACTTTCCACTAGAA	56
*ITGB5*	F: GGAGCCAGAGTGTGGAAACA R: GAAACTTTGCAAACTCCCTC	57
*CD44*	F: ATAATTGCCGCTTTGCAGGTGTATT R: ATAATGGCAAGGTGCTATTGAAAGCCT	60
*HAS2*	F: TATCACTGCTGCTGGGACTTGAAC R: AACACTTTCAGGCGGATGCAC	60
*RHAMM*	F: AGAACCAACTCAAGCAACAGG R: AGGAGACGCCACTTGTTAATTTC	60

## Data Availability

The raw data supporting the conclusions of this article will be made available by the corresponding authors on request.

## Abbreviations

The following abbreviations are used in this manuscript:

2D	Two-dimensional
3D	Three-dimensional
BC	Breast cancer
cDNA	Complementary DNA
CD44	Cluster of differentiation 44
CI	Confidence interval
Col I	Collagen type I
Ct	Cycle threshold
DMEM	Dulbecco’s modified Eagle’s medium
ECM	Extracellular matrix
EDTA	Ethylenediaminetetraacetic acid
ERα	Estrogen receptor alpha
ERβ	Estrogen receptor beta
shERβ	Estrogen receptor beta-suppressed
FBS	Fetal bovine serum
FN	Fibronectin
GAG	Glycosaminoglycan
HA	Hyaluronan
HAS2	Hyaluronan synthase 2
HEPES	4-(2-hydroxyethyl)-1-piperazineethanesulfonic acid
HER2	Human epidermal growth factor receptor 2
HR	Hazard ratio
ITGA1	Integrin subunit alpha 1
ITGA2	Integrin subunit alpha 2
ITGAV	Integrin subunit alpha V
ITGB1	Integrin subunit beta 1
ITGB3	Integrin subunit beta 3
ITGB5	Integrin subunit beta 5
NaOH	Sodium hydroxide
NTC	Non-template control
PBS	Phosphate-buffered saline
PR	Progesterone receptor
qPCR	Quantitative polymerase chain reaction
RFS	Relapse-free survival
RHAMM	Receptor for hyaluronan-mediated motility
RNA	Ribonucleic acid
rRNA	Ribosomal RNA
SD	Standard deviation
sHA	Sulfated hyaluronan
TME	Tumor microenvironment
TNBC	Triple-negative breast cancer
